# Perfusion and diffusion-weighted imaging parameters: Comparison between pre- and postbiopsy MRI for high-grade glioma

**DOI:** 10.1097/MD.0000000000030183

**Published:** 2022-09-02

**Authors:** Ryo Kurokawa, Akira Baba, Mariko Kurokawa, Aristides Capizzano, Yoshiaki Ota, John Kim, Ashok Srinivasan, Toshio Moritani

**Affiliations:** a Division of Neuroradiology, Department of Radiology, University of Michigan, Ann Arbor, MI.

**Keywords:** diffuse midline glioma, diffusion-weighted imaging, dynamic susceptibility contrast, high-grade glioma, perfusion

## Abstract

We aimed to evaluate the differences in dynamic susceptibility contrast (DSC)- magnetic resonance imaging (MRI) and diffusion-weighted imaging (DWI) parameters between the pre- and postbiopsy MRI obtained before treatment in patients with diffuse midline glioma, H3K27-altered. The data of 25 patients with pathologically proven diffuse midline glioma, H3K27-altered, were extracted from our hospital’s database between January 2017 and August 2021. Twenty (median age, 13 years; range, 3–52 years; 12 women) and 8 (13.5 years; 5–68 years; 1 woman) patients underwent preoperative DSC-MRI and DWI before and after biopsy, respectively. The normalized corrected relative cerebral blood volume (ncrCBV), normalized relative cerebral blood flow (nrCBF), and normalized maximum, mean, and minimum apparent diffusion coefficient (ADC) were calculated using the volumes-of-interest of the tumor and normal-appearing reference region. The macroscopic postbiopsy changes (i.e., biopsy tract, tissue defect, and hemorrhage) were meticulously excluded from the postbiopsy measurements. The DSC-MRI and DWI parameters of the pre- and postbiopsy groups were compared using the Mann–Whitney *U* test. The ncrCBV was significantly lower in the postbiopsy group than in the prebiopsy group [prebiopsy group: median 1.293 (range, 0.513 to 2.547) versus postbiopsy group: 0.877 (0.748 to 1.205), *P* = .016]. No significant difference was observed in the nrCBF and normalized ADC values, although the median nrCBF was lower in the postbiopsy group. The DSC-MRI parameters differed between the pre- and postbiopsy MRI obtained pretreatment, although the macroscopic postbiopsy changes were carefully excluded from the analysis. The results emphasize the potential danger of integrating and analyzing DSC-MRI parameters derived from pre- and postbiopsy MRI.

## 1. Introduction

MRI is an essential noninvasive modality for diagnosis, biopsy/surgical/radiation planning, and evaluation of the therapeutic effects in patients with glioma. Dynamic susceptibility contrast (DSC) is a contrast-enhanced perfusion-weighted MRI method that plays an important role in the grading of glioma, treatment effect prediction, and evaluation of disease progression. The cerebral blood volume (CBV) and cerebral blood flow (CBF) derived from DSC-MRI differ significantly between high-and low-grade gliomas.^[1,2]^ Choi et al^[3]^ demonstrated the utility of DSC-MRI and dynamic contrast-enhanced MRI as predictive/prognostic imaging biomarkers in patients with recurrent glioblastoma treated with bevacizumab. Perfusion MRI is also considered to be useful for the determination of the biopsy site.

Diffusion-weighted imaging (DWI) is another unique MRI sequence that facilitates noninvasive observation of the microstructure of tumors and surrounding brain tissue. DWI parameters, especially the apparent diffusion coefficient (ADC), have also been shown to be important for the management of glioma.^[4–7]^ For example, ADC values derived from DWI-MRI with b values of 1000 s/mm^2^ or 3000 s/mm^2^ obtained prior to treatment can help differentiate between high- and low-grade gliomas with high sensitivity and specificity.^[8]^ Recurrent glioblastomas with low ADC were shown to be associated with shorter progression-free survival in patients treated with bevacizumab.^[7]^ DWI and perfusion MRI are considered useful for the determination of the biopsy site.^[9–11]^

The clinical practice guidelines for the diagnosis, treatment, and follow-up provided by the European Society for Medical Oncology^[12]^ stipulate that tissue diagnosis is mandatory for high-grade gliomas. Stereotactic biopsy remains the mainstay for tissue sampling, owing to its high diagnostic success rate that exceeds 95%.^[13,14]^ On the other hand, biopsy is associated with the risk of various complications, including morbidity (6.7%),^[13]^ hemorrhage (4.7%),^[15]^ and mortality (0.6%).^[13]^ Biopsy may induce local changes such as biopsy tract formation, tissue defect, and hemorrhage, which can affect the results of neuroradiological studies. However, previous studies that analyzed pretreatment MRI failed to specify whether MRI was acquired prebiopsy. Therefore, it is possible that these studies analyzed the results of pre- and postbiopsy MRI simultaneously. Meanwhile, the changes that may or may not occur in the DSC-MRI and DWI parameters after biopsy have not been established, especially when measurements are acquired without taking the macroscopic postbiopsy changes into account.

Therefore, the purpose of this study was to evaluate the differences in the DSC-MRI and DWI parameters of patients with diffuse midline glioma, H3K27-altered (for which biopsy is essential for diagnosis and treatment determination) between the pre- and postbiopsy MRI scans obtained before treatment while attempting to exclude the macroscopic postbiopsy changes from the analysis.

## 2. Materials and Methods

Institutional review board approval was obtained for the conduct this study, which exempted the study from the requirement to acquire patient consent. Data were acquired in compliance with all applicable Health Insurance Portability and Accountability Act regulations and were de-identified before all analyses.

### 2.1. Patients

We searched the electronic database of our hospital and found a total of 68 patients with pathologically proven diffuse midline gliomas, H3K27-altered between January 2017 and August 2021.

The exclusion criteria were as follows:

- The tumors were not pathologically proven diffuse midline gliomas, H3K27-altered.
- DSC-MRI was not performed before treatment.
- Extracranial tumors.

The H3 alteration status was identified using immunohistochemistry with antibodies specific to the H3K27M mutation or detecting the fusion gene by gene sequencing. Forty-three of the 68 patients were excluded according to the following exclusion criteria: DSC-MRI was performed with posttreatment MRI (n = 26); DSC-MRI was not performed (n = 14), extracranial tumors (n = 2), and severe artifact (n = 1). Finally, 25 patients were included for further evaluation in this study. These patients underwent both pre- and postbiopsy DSC-MRI (n = 3), prebiopsy DSC-MRI alone (n = 17), and postbiopsy DSC-MRI alone (n = 5).

### 2.2. MRI scanning protocol

The MRI scanning protocol is summarized in Table 1. Brain MRI was performed using 1.5-T (n = 11) and 3-T (n = 17) MRI devices (Ingenia, Achieva: Philips Healthcare, Eindhoven; MAGNETOM Vida: Siemens, Munich) with a 32-channel head coil in the supine position. An intravenous bolus of 20 mL of gadoteridol (ProHance, Bracco Diagnostics, Inc., Princeton, NJ) or gadobenate dimeglumine (Multihance, Bracco diagnostics, Singen, Germany) was administered for DSC-MRI using a power injector through a peripheral arm vein with a flow rate of 5.0 mL/s, followed by flushing with 20 mL of saline. The patients were administered an additional 5 mL of the contrast medium 5 minutes prior to the dynamic perfusion scan. The parameters of the fast field echo T2*-weighted imaging were as follows: plane, axial; repetition time, 1500–1850 ms; echo time, 30 to 50 ms; number of excitations, 1; slice thickness, 4–5 mm; slice increment, 5 to 5.2 mm; field-of-view, 220 to 230 mm; matrix, 128 × 128–144 × 144; dynamic measurements, 40; temporal resolution, 1.5 s; and total acquisition time, 1 minute 4.5 s. The acquisition parameters for DWI were as follows: plane, axial; repetition time, 4000–6600 ms; echo time, 58.2 to 101.5 ms; number of excitations, 1 or 2; slice thickness, 4–5 mm; slice increment, 4.4 to 5 mm; field-of-view, 220 to 240 mm; matrix, 240 × 240–320 × 320; b-value = 0 and 1000 s/mm^2^.

**Table 1:** MRI acquisition protocol.

	Fat-suppressed T2WI	Fluid-attenuated inversion recovery	precontrast T1WI	postcontrast T1WI	postcontrast fsT1WI	DWI(b = 0, 1000 s/mm^2^)	Fast field echo T2*WI	SWI
Plane	Axial	Axial	Axial	Axial	Axial	Axial	Axial	Axial
Repetition time (ms)	3000–5000	8500–11,000	500–2000	500–650	550–2440	4000–6600	1500–1850	27–52
Echo time (ms)	80–105	105–140	9–20	10.2–15	9–13	58.2–101.5	30–50	20
Flip angle (degree)	90–132	90–150	69–150	69–90	69–150	90–180	40–90	15
Number of excitations	1–3	1,2	1,2	1	2	1,2	1	1
Slice thickness/ increment (mm)	4–5/4.4–6	4–5/4.4–6	4–5/4.4–6	4–5/4.4–6	4–5/4.4–6	4–5/4.4–5	4–5/5–5.2	2/2
Field-of-view (mm)	220–230	220–230	230	230	220–230	220–240	220–230	220–230
Matrix	448 × 448–672 × 672	320 × 310–560 × 560	320 × 320–576 × 576	384 × 384–560 × 560	320 × 320–352 × 352	240 × 240–320 × 320	128 × 128–144 × 144	256 × 232

T2WI = T2-weighted imaging, T1WI = T1-weighted imaging, DWI = diffusion-weighted imaging, T2

*WI = T2

*-weighted imaging, SWI = susceptibility-weighted imaging

### 2.3. Quantitative DSC-MRI analyses

**Figure 1.**
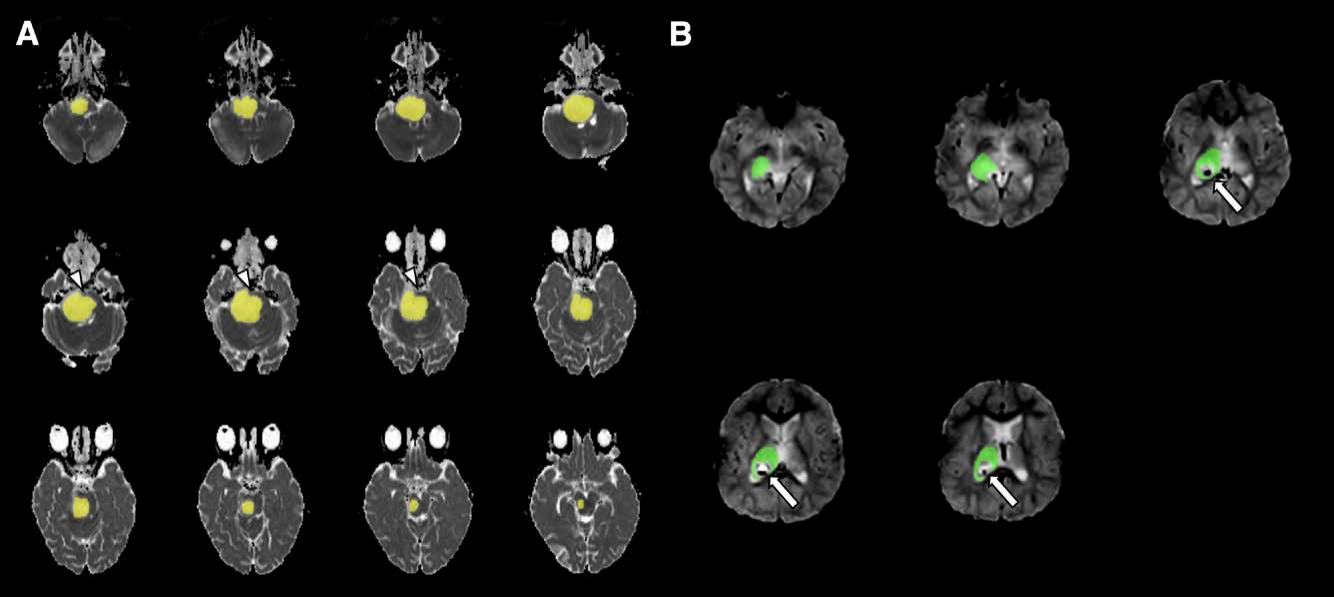
Example of VOI measurement. The vertebral artery was avoided in the ADC map in the prebiopsy scan (a, arrowheads). The postbiopsy change was avoided in the perfusion map in the postbiopsy scan (b, arrows). VOI = volume-of-interest, ADC = apparent diffusion coefficient.

Quantitative DSC-MRI analyses were conducted using OleaSphere (Version 3.0; Olea Medical, La Ciotat, France). The DSC-MRI data were processed with motion artifact correction using rigid-body registration. The arterial input function (AIF) was calculated automatically using cluster analysis techniques, and deconvolution of the AIF was performed with a time-insensitive block-circulant singular-value decomposition.^[16]^ Whole-brain corrected relative CBV (rCBV) and relative CBF (rCBF) maps were generated using voxel-wise division of the area under the concentration-time curve by the area under the AIF. One board-certified radiologist with 9 years of experience in neuroradiology carefully delineated the regions-of-interest (ROIs) freehand on every axial slice of the perfusion maps depicting the tumor to generate the volumes-of-interest (VOIs) while excluding cystic, hemorrhagic, or necrotic regions and vessels using reference T2WI, fluid-attenuated inversion recovery images, pre- and postcontrast T1WI, T2*WI, and/or susceptibility-weighted images under the direct supervision of another board-certified radiologist with 13 years of experience in neuroradiology. Biopsy tracts, tissued defects, and hemorrhagic changes were also carefully avoided during postbiopsy examinations by referring to the conventional sequences and prebiopsy MR images, if applicable. Another ROI was placed over the normal-appearing contralateral head of the caudate nucleus as a reference to correct for age- and patient-dependent variations in perfusion parameters.^[2]^ The VOI and reference ROI were transposed to the corrected rCBV and rCBF maps. Finally, normalized corrected rCBV (ncrCBV) and normalized rCBF (nrCBF) were calculated by dividing the mean corrected rCBV and mean rCBF of the tumor by those of the reference regions. An example of VOI measurement is demonstrated in Figure 1.

### 2.4. Quantitative ADC analyses

DWI was performed during every MRI examination with DSC-MRI. ADC maps were constructed using a mono-exponential fitting model using OleaSphere. The VOIs were generated on the solid components of the tumors using the same method that was used for DSC-MRI analysis. Another ROI was placed as a reference in the normal-appearing posterior limb of the internal capsule, while avoiding the border areas.^[17]^ The normalized maximum (nADC_max_), mean (nADC_mean_), and minimal ADC (nADC_min_) were calculated for each ROI of the tumors by dividing ADC values by the reference mean ADC.

### 2.5. Statistical analysis

The DSC-MRI parameters (nrCBF and ncrCBV) and ADC values (nADC_max_, nADC_mean_, and nADC_min_) were compared between the pre- and postbiopsy groups using the Mann–Whitney *U* test. The parameters of pre- and postbiopsy MRI were not compared for the same patient due to the small sample size (n = 3). All statistical analyses were performed using R software (version 4.0.0; R Foundation for Statistical Computing, Vienna, Austria).

## 3. Results

The study population consisted of 20 patients who underwent prebiopsy DSC-MRI (median age, 13 years; range, 3–52 years; 12 women), including 3 patients who underwent both pre- and postbiopsy DSC-MRI (6, 13, and 14 years of age; 1 woman) and 8 patients who underwent postbiopsy DSC-MRI (median age, 13.5 years; range, 5–68 years; 1 woman). The median duration between the brain biopsy and postbiopsy MRI was 12.5 days (range, 3–107 days). Hemorrhage was detected at the biopsy site in two patients (25.0%).

### 3.1. DSC-MRI parameters

The results of DSC-MRI analyses are summarized in Table 2. A pulsed input pattern was observed in the AIF curves in all patients. The ROIs were placed successfully over the solid components of the tumors, while avoiding visual image changes caused by biopsy for the postbiopsy group.

**Table 2:** Comparison of the DSC-MRI and DWI parameters between the pre- and postbiopsy groups.

Prebiopsy group (n = 20)	Corrected rCBV_tumor	rCBF_tumor	Corrected rCBV_ref	rCBF_ref	ncrCBV	nrCBF	ADC_max_	ADC_mean_	ADC_min_	ADC_mean__ref	nADC_max_	nADC_mean_	nADC_min_
Median	3.190	29.480	2.210	28.650	1.293	0.948	2.220	1.235	0.735	0.758	2.777	1.612	0.961
Maximum	8.320	65.090	5.260	86.290	2.547	2.269	3.070	1.900	1.080	0.990	4.488	2.568	1.367
Minimum	0.610	8.100	1.190	16.880	0.513	0.426	1.256	0.770	0.410	0.680	1.794	1.085	0.485
Postbiopsy group (n = 8)	Corrected rCBV_tumor	rCBF_tumor	Corrected rCBV_ref	rCBF_ref	ncrCBV	nrCBF	ADC_max_	ADC_mean_	ADC_min_	ADC_mean__ref	nADC_max_	nADC_mean_	nADC_min_
Median	1.825	20.605	1.970	22.530	0.877	0.811	1.805	1.150	0.645	0.740	2.654	1.582	0.903
Maximum	3.130	31.780	3.610	39.340	1.205	1.186	2.430	1.260	0.700	0.840	3.038	1.877	1.046
Minimum	1.040	11.410	1.390	14.770	0.748	0.507	1.020	0.860	0.580	0.650	1.397	1.178	0.714
*P*-value	0.022*	0.099	0.52	0.099	0.016*	0.82	0.23	0.31	0.54	0.70	0.33	0.44	0.41

DSC = dynamic susceptibility contrast, DWI = diffusion-weighted imaging, ncrCBV = normalized corrected relative cerebral blood volume, nrCBF = normalized relative cerebral blood flow, nADC_max/mean/min_ = normalized maximum/mean/minimum apparent diffusion coefficient, ref = reference.

**Figure 2.**
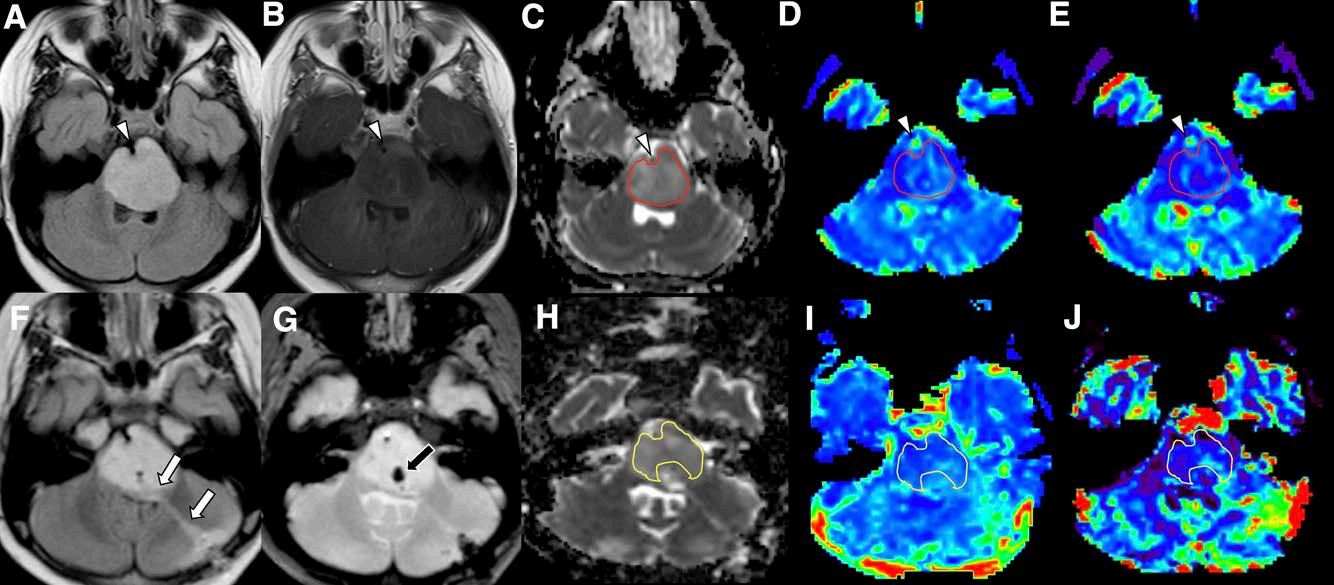
Example of region-of-interest (ROI) placement (a–e: prebiopsy MRI, f–j: postbiopsy MRI) On the prebiopsy MRI, the expansile pontine diffuse midline glioma, H3K27-altered appears hyperintense on the FLAIR image (a) with scarce enhancement on the postcontrast T1-weighted image (b). ROIs are placed on the ADC map (c), corrected rCBV map (d), and rCBF map (e) while avoiding the basilar artery (arrowheads). the biopsy tract (f, white arrows) and hemorrhagic focus (g, black arrow) are observed on the postbiopsy FLAIR MRI image (f) and T2*-weighted image (g). ROIs are placed on the ADC map (h), corrected rCBV map (i), and rCBF map (j) while avoiding basilar artery and the postbiopsy changes. DSC = dynamic susceptibility contrast, ADC = apparent diffusion coefficient, FLAIR = fluid-attenuated inversion recovery, rCBV = relative cerebral blood volume, rCBF = relative cerebral blood flow.

The ncrCBV was significantly lower in the postbiopsy group compared to the prebiopsy group [prebiopsy group: median 1.293 (range, 0.513 to 2.547) versus postbiopsy group: 0.877 (0.748 to 1.205), *P* = .016]. Although the median nrCBF and rCBF were lower in the postbiopsy group and the rCBF approached significance, the difference between the two groups was not statistically significant [prebiopsy group: median 0.948 (range, 0.426 to 2.269) versus postbiopsy group, 0.811 (0.507 to 1.186), *P* = .82]. The representative MRI scans obtained pre- and postbiopsy are shown in Figure 2.

### 3.2. ADC values

The results of ADC analyses are summarized in Table 2. No significant difference was observed between the pre- and postbiopsy groups [prebiopsy group: nADC_max_, median: 2.777 (range, 1.794 to 4.488) versus postbiopsy group: 2.654 (1.397 to 3.038), *P* = .33; nADC_mean_, median: 1.612 (range, 1.085 to 2.568) versus 1.582 (1.178 to 1.877), *P* = .44; nADC_min_, median: 0.961 (range, 0.485 to 1.367) versus 0.903 (0.714 to 1.046), *P* = .41].

## 4. Discussion

In this study, we investigated the differences in the DSC-MRI and DWI parameters in H3K27-altered diffuse midline glioma between the pre- and postbiopsy MRI scans, while avoiding the macroscopic postbiopsy changes during measurement. There was a significant decrease in the ncrCBV in the postbiopsy group compared to the prebiopsy group [prebiopsy group, median: 1.293 (range, 0.513 to 2.547) versus postbiopsy group, median: 0.877 (0.748 to 1.205), *P* = .016]. The nrCBV and DWI parameters did not differ significantly between the two groups.

The treatment of glioma, especially high-grade glioma, entails multidisciplinary management including surgery, radiotherapy, chemotherapy, molecular targeted therapy, and novel therapies such as oncolytic virus therapy.^[18]^ Pathological diagnosis is mandatory for appropriate treatment planning, and stereotactic biopsy remains the mainstay owing to its high diagnostic success rate,^[13,14]^ despite the risk of complications such as hemorrhage, seizure, infection, morbidity, and mortality.^[15,19,20]^

MRI is an essential noninvasive imaging modality for the diagnosis and planning for biopsy in patients with glioma. Studies have demonstrated the important role of advanced MRI sequences, including DSC-MRI and DWI, in tumor grading, treatment effect prediction, and evaluation of disease progression in patients with glioma.^[1–8,21–24]^ The protocol employed by these studies included pretreatment MRI alone, posttreatment MRI alone, and both pre- and posttreatment MRI. Studies that analyzed pretreatment MRI did not often specify whether MRI acquisition was performed before biopsy. Moreover, the changes occurring before and after biopsy prior to treatment have not yet been illuminated. Therefore, the validity of results obtained by combining the findings of pretreatment, and pre- and postbiopsy DSC-MRI and DWI parameters for the assessment of the diagnosis and survival remains unknown. Although we carefully avoided the macroscopic changes induced by biopsy (i.e., biopsy tract, tissue defect, and hemorrhagic change) in this study, the results showed a significant decrease in the ncrCBV, which indicates the importance of analyzing the MRI findings separately, especially the DSC-MRI parameters, before and after biopsy.

The mechanism underlying the decrease in the ncrCBV in the present study could not be clarified owing to the lack of pathological data. The rCBV, which reflects the volume of blood in a given amount of tissue, is known to be positively correlated with tumor vessel density^[25]^ and is thus significantly higher in high-grade gliomas than that in low-grade gliomas.^[1,2,26]^ The decrease in the rCBV on postbiopsy MRI can be explained by the occurrence of intratumoral edema and small-vessel injury, resulting in a decrease in capillary perfusion. In the present study, DSC-MRI acquisition was performed using fast field echo T2*-weighted imaging, which enabled the evaluation of tissue perfusion by depicting the transient change in the magnetic susceptibility caused by the passage of contrast material as a signal drop. Therefore, intratumoral hemorrhage caused by biopsy could affect the DSC-MRI parameters, albeit to a limited extent, since we carefully excluded hemorrhagic changes from the measurement by referring to the conventional MRI sequences, including the T2*-weighted and susceptibility-weighted images. On the other hand, significant differences were found between the nrCBF of the pre- and postbiopsy groups, although the median nrCBF and rCBF were lower and the rCBF approached significance in the postbiopsy group. There is a possibility that the difference in the nrCBF could have been underestimated due to the small sample size.

There are some limitations to this study. First, it was a single-institutional retrospective study with a small sample population. Second, the range of the patient ages (3–68 years) and the time range between brain biopsy and postbiopsy DSC-MRI (3–107 days) could have induced heterogeneity in the patients. Further studies with a more homogeneous study population will be desired. Third, pathological data associated with the postbiopsy changes were not available. However, given the risk of complications associated with stereotactic biopsy, performing multiple biopsies within a short time is not practical in routine clinical practice. Therefore, it is necessary to accumulate cases from multiple institutions, which may be challenging, for a more detailed pathological study of the postbiopsy site.

In conclusion, the ncrCBV was significantly lower in patients with H3K27-altered diffuse midline glioma in the postbiopsy group compared to the prebiopsy group, while the nrCBF and nADC_max/mean/min_ did not differ significantly between the two groups. The results emphasize the potential danger of integrating and analyzing DSC-MRI parameters derived from pre- and postbiopsy MRI.

## Author contributions

Conceptualization: Ryo Kurokawa

Data curation: Ryo Kurokawa, Akira Baba

Formal analysis: Ryo Kurokawa

Investigation: Ryo Kurokawa, Akira Baba

Methodology: Ryo Kurokawa, Akira Baba, Mariko Kurokawa, Aristides Capizzano

Project administration: John Kim

Supervision: Ashok Srinivasan, Toshio Moritani

Validation: Toshio Moritani

Writing (original draft): Ryo Kurokawa

Writing (review & editing): Akira Baba, Mariko Kurokawa, Aristides Capizzano, Yoshiaki Ota,

John Kim, Ashok Srinivasan, Toshio Moritani
